# Molecular population genetics of male and female mitochondrial genomes in subarctic *Mytilus trossulus*

**DOI:** 10.1007/s00227-013-2223-7

**Published:** 2013-04-04

**Authors:** Beata Śmietanka, Małgorzata Zbawicka, Tomasz Sańko, Roman Wenne, Artur Burzyński

**Affiliations:** Department of Genetics and Marine Biotechnology, Institute of Oceanology Polish Academy of Sciences, Powstańców Warszawy 55, 81-712 Sopot, Poland

## Abstract

The doubly uniparental inheritance system allows for the use of two independent mitochondrial genomes for population history investigations. Under this system, two lineages of mitochondrial DNA (mtDNA) exist and males are typically heteroplasmic, having the additional, usually divergent, mitochondrial genome inherited from their male parent. This additional mtDNA typically evolves faster, potentially allowing for insight into more recent events in population history. Few studies did explore this possibility in marine mussels *Mytilus* showing its usefulness. Recent observations of the *Mytilus trossulus* mussels who have retained their native mtDNA in European waters posed the question of their origin. Are they part of a population present, but previously undetected, or is this a potentially human mediated, ongoing spread of an invasive species? To tackle this question, we amplified with species-specific primers and sequenced an approximately 1,200-bp-long fragment spanning *COIII* and *ND2* genes from both mitochondrial genomes of mussels sampled at five locations worldwide, covering the whole *M. trossulus* range. The overall pattern of polymorphisms is compatible with the entirely postglacial history of the whole species, indicating a very deep bottleneck at last glacial maximum, with possible retention of the whole species in a single refugium, and the effective population size of no more than a few thousands. Both analyses of molecular variance and isolation with migration (IM) models point at the West Atlantic as the source of the European *M. trossulus* mussels, at least the ones who retained their native mtDNA. The hypothesis that this is an ongoing, human-mediated process was considered. To this end, comparison with the well-known case: the introduction of congeneric mussel, *Mytilus galloprovincialis,* from Mediterranean Sea to Asia was used. This introduction occurred within the last 100 years. The results inferred by the IM model suggest that the timing and structure of transatlantic migration of *M. trossulus* differs significantly from the *M. galloprovincialis* case: it is more than 1,000 years old and involves a much larger fraction of the ancestral population. Therefore, most likely, this invasion is not a human-mediated process.

## Introduction

The smooth-shelled blue mussel *Mytilus edulis* species complex consists of three recognised, closely related and hybridising members: *M. edulis*, *Mytilus galloprovincialis* and *Mytilus trossulus* (McDonald et al. 1991; Gosling 1992). Some authors consider the Chilean mussel *M. edulis platensis* a separate species *Mytilus chilensis* (Borsa et al. 2011), bringing the total number of species in this complex to four. The next related mussel species: *Mytilus californianus* and *Mytilus coruscus* do not hybridise with other forms and are usually not considered part of the complex.

*Mytilus trossulus* originated in the Pacific about 3.5 million years ago (Rawson and Hilbish 1995b) and subsequently colonised the North Atlantic after the Bering Strait opening, where it gave rise to *M. edulis* and *M. galloprovincialis* (Riginos and Cunningham 2005). There must have been at least one more invasion of Pacific mussels into the Atlantic as the presence of all three members of the complex in European waters was confirmed by allozyme and DNA markers (Varvio et al. 1988; Gosling 1994; Śmietanka et al. 2004). In European waters, *M. trossulus* generally settles in lower salinity waters than its congeners: it was identified in the Baltic Sea (Väinölä and Hvilsom 1991), in Norwegian fjords near Bergen (Ridgway and Nævdal 2004) and recently in Scotland (Beaumont et al. 2008; Dias et al. 2008; Zbawicka et al. 2010) and Barents Sea (Väinölä and Strelkov 2011). Single mussels with *M. trossulus* alleles were also found in the Netherlands (Śmietanka et al. 2004). At the western coast of the North Atlantic, the southern range of *M. trossulus* ends in the Gulf of Maine. In North America, as well as in Asia, the species is widely distributed along Pacific coasts. In the areas where the ranges of two *M. edulis* complex species overlap, the hybridisation may occur. The well-known European hybridisation areas occur between *M. edulis* and *M. trossulus* in the Baltic Sea (Riginos and Cunningham 2005) and Scotland waters (Beaumont et al. 2008; Zbawicka et al. 2010).

*Mytilus* spp. have an unusual system of mitochondrial DNA (mtDNA) transmission known as doubly uniparental inheritance (DUI). Under DUI, males are heteroplasmic, having an additional mtDNA (M genome) inherited from their fathers, in addition to the typical mtDNA inherited from their mothers and in this case called the F genome (Zouros et al. 1994; Skibinski et al. 1994b). The sequence divergence between M and F genomes in *Mytilus* approaches 30 %. It has been noted that the M genome evolves faster than the F genome (Skibinski et al. 1994a; Stewart et al. 1995; Rawson and Hilbish 1995a). This observation was discussed in the context of postulated relaxed selection within the M lineage (Stewart et al. 1996). This, in theory, should make the M genome sequences more suitable for investigating genetic structure of mussel populations. In practice, however, in few cases when both M and F genome sequences were used, either no genetic structure was found whatsoever—in case of *M. californianus* (Ort and Pogson 2007), or, paradoxically, the F genome sequences provided better geographical resolution—in *M. edulis*/*M. galloprovincialis* case (Śmietanka et al. 2009).

The apparently recent expansion of *M. trossulus* in Scotland (Beaumont et al. 2008; Dias et al. 2008; Zbawicka et al. 2010) raised the question of the origin of the invading mussels. In order to identify their origin, we investigated *M. trossulus* on the global scale, with populations from both oceans sampled, using mitochondrial markers from both genomes. This phylogeographical analysis gave insight into the timing and mode of evolution as well as demographic history of the species.

## Materials and methods

### Sample collection

Samples of blue mussels *M. trossulus* were collected from 5 localities: the west coast of the Japan Sea (JSE); Howe Sound, Canada (VAN); Nova Scotia, Canada (NSC); Loch Etive, Scotland (LET) and the Aleutian Islands (ALE). Samples were collected in years 2007 and 2011, except for NSC, which was sampled in 1997. The sample of *M. galloprovincialis,* used as the reference in some analyses, was collected in South Korea near Busan (BUS), in 2006. Approximately 30–60 individuals per each sample were initially taken. Mussels were taxonomically identified using nuclear DNA markers: *Me15*/*16* (localised in adhesive protein gene) (Inoue et al. 1995), ITS (RFLP marker comprising *ITS*-*1*, 5.8S and *ITS*-*2* regions of rDNA (Heath et al. 1995) and EFbis (RFLP marker localised in an intron of elongation factor 1α gene) (Bierne et al. 2003; Kijewski et al. 2006). Only pure *M. trossulus* specimens were taken for further analysis, except for the BUS sample. Mussels were sexed by microscopic examination of the mantle. Sex determination was possible for approximately half of the samples only. Prior to DNA extraction, samples were preserved in 96 % ethanol or stored at −70 °C.

### DNA extraction and amplification

Small pieces of the mantle tissue were removed, homogenised, and DNA was extracted using the plant CTAB method (Doyle and Doyle 1987) modified for animal tissues by inclusion of proteinase K in the extraction buffer by Hoarau et al. (2002). DNA was suspended in sterile-filtered distilled water. The fragment of mtDNA spanning 3′ part of *ND2*, two tRNA genes: *tRNA* ^*SER*^ and *tRNA* ^*MET*^ and 5′ part of *COIII* gene was PCR amplified separately from M and F genome. Specific primers were designed based on the published complete M and F genome sequences from *M. trossulus* (Śmietanka et al. 2010; Zbawicka et al. 2010). For F genome amplification, the following primers were used: the F-specific F1T: TTCCTAGTGCAACTTCGAGAATA and the universal U2T: AAGGAAAGGAGGCATCCC. For M genome amplifications, the M-specific primer M1T: CCGAACCCTTCCTCTACAAG was used, along with the U2T primer. The PCR followed the protocol established for amplification of the same region from *M. edulis* and *M. galloprovincialis* (Śmietanka et al. 2009); the new primers were designed to be compatible with the same reaction conditions. The lengths of the amplified products were approximately 1,280 bp for the F and 1,420 bp for the M genomes. For the *M. galloprovincialis* sample, the previously described primers were used (Śmietanka et al. 2009). Products of amplification were separated by 1 % agarose gel electrophoresis in a 0.5× TBE buffer and visualised with ethidium bromide in UV light. All PCR products were purified by alkaline phosphatase and exonuclease I treatment (Werle et al. 1994) and sequenced with the BigDye™ terminator cycle sequencing method. An ABI 3730 automatic sequencer was used to separate reaction products. A total of 139 sequences for the F genome and 83 for the M genome were obtained from *M. trossulus*. All sequences have been deposited in GenBank under accession numbers KC565896–KC566117, KC800995-KC801023.

### Bioinformatic analysis

Obtained sequences were assembled using the Gap4 program from Staden Package version 1.7.0 (Staden et al. 2001) and aligned using ClustalX version 1.83 (Thompson et al. 1997). After trimming the beginnings and endings of the sequences, the length polymorphism was minimal: all F sequences were 1,171 bp long, and most of the M sequences were 1,304 bp long with few sequences up to two bp shorter or longer than that (1,302–1,306 bp). In order to detect potential recombination signals that could interfere with the phylogenetic analysis, the RDP suite of programs with the default settings (Martin et al. 2005) was used. As no recombination signal was detected, all obtained sequences were taken for further analysis. Standard diversity indices, such as the number of segregating sites (S), haplotype diversity (HD), *θ* per site, nucleotide diversity (*π*) and Tajima’s *D,* were calculated using DnaSP version 5.10 (Librado and Rozas 2009). The coding parts of nucleotide sequences were used to estimate the nucleotide diversity in synonymous (*K*_S_) and nonsynonymous (*K*_A_) sites (Zhang et al. 2006). Drosophila mtDNA genetic code (NCBI translation Table 5) was used whenever necessary after Hoffmann et al. (1992). For assessing the genetic structure at intra- and interpopulation level for both M and F mtDNA lineages, hierarchical analysis of molecular variance (AMOVA) was performed in ARLEQUIN version 3.5.1.3 (Excoffier and Lischer 2010). The haplotype distance matrix was calculated in ARLEQUIN and taken into account in estimates of *F* statistics. The statistical significance of obtained indices (Φ_ST_, Φ_SC_, Φ_CT_) was assessed by 1,023 permutations of the original data matrix following the Bonferroni adjustment (Rice 1989). Because samples were collected in different years, an AMOVA analysis was run with year specified as a variable. The year of sample collection did not contribute to the observed polymorphism (*P* > 0.05). The neighbour-joining trees illustrating the genetic relatedness of studied samples were constructed in MEGA version 5.0 (Tamura et al. 2011), based on the matrix of genetic distances calculated in ARLEQUIN. Phylogenetic relationships between sequences were reconstructed using the maximum likelihood (ML) method. To find the best-fit model of sequence evolution, jModelTest version 0.1.1 (Posada 2008) was applied. The TIM3+G model for the F data set was selected by Akaike (AIC) as well as Bayesian (BIC) information criterion. For the M data set, the TIM3+G was recommended by BIC and GTR+G substitution model was selected by AIC. The models were used for both mtDNA loci, respectively, to construct the trees using PhyML v. 3.0 software (Guindon and Gascuel 2003). The trees were tested for significance by bootstrapping (500 pseudoreplicates). For highly polymorphic intrapopulation, mitochondrial markers estimation of classic phylogenetic trees is often not appropriate due to inevitable uncertainties of the relationships between haplotypes. This relationship could be better solved by estimating a network of haplotypes connected by a minimal number of mutational steps. To this end, Network software version 4.6.1.0 was used (http://www.fluxus-engineering.com). This software implements the median-joining algorithm to reconstruct the network (Bandelt et al. 1995). Different settings for the homoplasy level parameter, *ε*, were tested, and *ε* = 30 was eventually used. To account for differences in substitution rates, the transversions were weighted twice as much as the transitions. The most parsimonious solutions (“Steiner trees”) of the MJ networks were inferred (Polzin and Daneshmand 2003).

The models allowing population isolation followed by restricted migration implemented in IM (Nielsen and Wakeley 2001) and IMa2 (Hey 2010) were used. Both use the Monte Carlo Markov chain approach to sample posterior distribution of genealogies, and therefore, it is critical to run them for a sufficiently long time and with good mixing to obtain meaningful results. Standard measures were taken to ensure that this was the case: each analysis was run with long burnin, in multiple replicates (at least 10), ensuring that all runs converged at the same solution. These time-consuming tasks were run on supercomputers in parallel, as recommended in the IMa2 manual. Effective sample sizes (ESS) of all measured indices were above 300.

To reconstruct the past demography, the Extended Bayesian Skyline Plot (EBSP) approach was used, as implemented in BEAST version 1.6.2 (Drummond and Rambaut 2007). Since the approach can be applied to unstructured populations only, the samples were either analysed separately or combined accordingly to AMOVA and IM results. The best models of molecular evolution for this analysis were selected by Bayes factor comparisons. Each analysis was run in quadruplicates to ensure the convergence on the global optimum; the runs were subsequently combined. To achieve the required ESS >300, the total length of the combined runs was approximately 200 million generations in all cases.

To test for departures from neutrality, the McDonald and Kreitman (M–K) test (McDonald and Kreitman 1991) was used. The test is based on the comparison of variation at synonymous (silent) and nonsynonymous (replacement) sites within and between lineages. Under the neutral theory, the ratio of sites polymorphic within one or more species/clades to the number of sites fixed between them must be equal for synonymous and nonsynonymous substitutions.

## Results

### Molecular diversity at intra- and interpopulation level

**Fig. 1:**
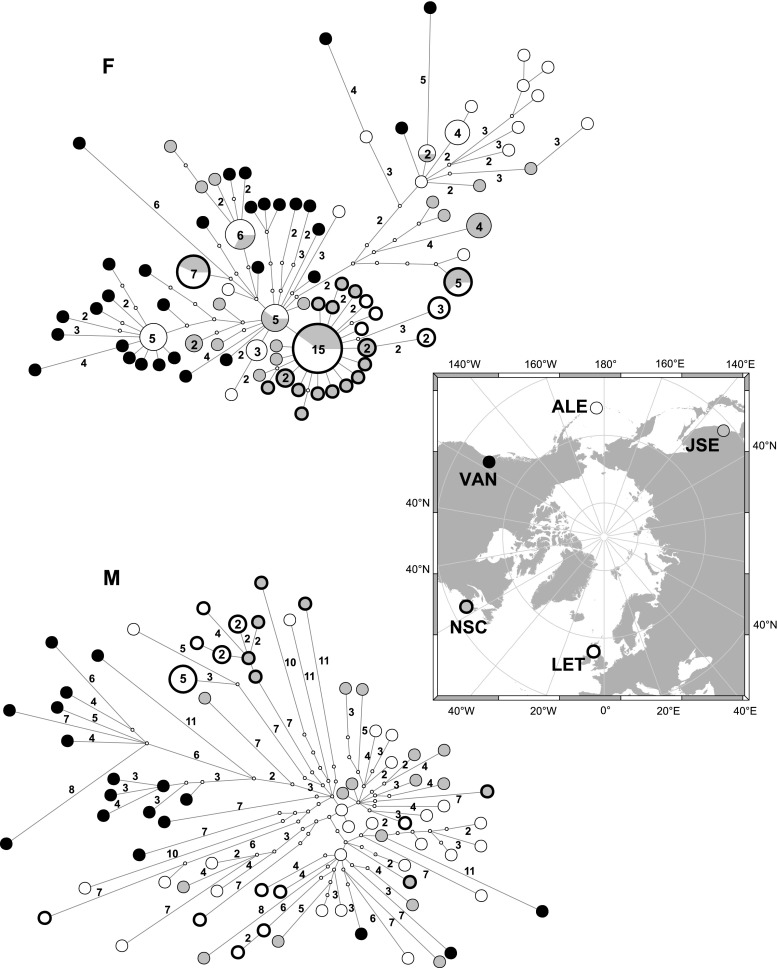
The representative examples of the shortest haplotype trees derived from MSNs generated by median-joining algorithm in network in the M and F data sets. Each *circle* represents a single haplotype with an area proportional to the number of observed individuals bearing the haplotype, additionally given as the *label inside* the *circles*. Singleton haplotypes are not labelled. *Small, open circles* represent median vectors inferred by the algorithm. They were used by alternative connections in the original network, which were either removed by the Stainer procedure (Polzin and Daneshmand 2003), or were absent from the presented example. *Numbers* on the *lines* connecting haplotypes indicate the number of mutational steps along each connection. Single step connections are not *labelled*. Individuals from the Atlantic have the *thicker line* around the *haplotype circle*. Individuals from JSE and NSC are in *grey*, the ones from LET and ALE are in *white* and the ones from VAN are in *black*. The exact location of each sampling site is shown in the centre

We examined 222 sequences from five localities of Pacific and Atlantic *M. trossulus* taxa. The F genome was represented by 139 sequences with the alignment length of 1,171 bp, whereas for the M genome, we obtained 83 sequences with the alignment length of 1,306 bp. We observed a significantly higher level of polymorphism for the M genome than for the F genome (Table 1). In the constructed ML phylogenetic, most of the subdivisions were supported by low and very low bootstrap values, without distinct clades in both F and M genome trees. Reliable support was obtained only in few cases supporting small intrapopulation clades with a few individuals only, indicating very shallow, young phylogeny (data not shown). To better investigate the relationship between obtained sequences, median-joining algorithm was applied to the whole data set (Fig. 1). The total number of identified haplotypes for the F genome was 87; for the M genome, there was a comparable number of 76 haplotypes even though a significantly smaller number of M sequences were obtained. Within the F group, the most frequent haplotype was present in 15 individuals, 14 additional haplotypes occurred in two or multiple copies and 72 of them were unique. In the case of the M group, one haplotype was common to five individuals, only three other occurred twice and the remaining 72 haplotypes were singlets. Although HD was very high (87–100 %) in both genomes, the total number of segregating sites and the average nucleotide diversity in the M sequences was twice as high as in the F sequences. Comparison of genetic diversity of mussels from the two oceans indicated higher genetic diversity in the Pacific than in the Atlantic. No clear reciprocal monophyly was observed between any of the samples in the data set—the haplotypes of various similarities were intermixed in all samples, producing a rather complex phylogeographical pattern. There were no cases of haplotypes shared between oceans, however. Within the F data set one, mostly Atlantic clade was noted, with clear star-like pattern indicating its recent expansion. However, two additional haplotypes were also present in the Atlantic at appreciable frequencies. Within the M data set, the only nonunique haplotypes were present in the LET sample, and all the remaining individuals in all samples had unique haplotypes with poorly resolved relationships. One small, exclusively Atlantic clade was observed, but 12 other Atlantic haplotypes were spread across the network without a clear pattern. Another relatively well-separated clade was composed of VAN sequences only, although some VAN sequences were also present outside that clade.

**Table 1:** Standard indices of genetic diversity in *ND2*–*COIII* region of the F and M *M. trossulus-*specific genomes for analysed populations

Samples	Genome	*N*	*S*	HD	*θ*	*π* (JC)	*D*_0_	*D*_4_
LET	F	20	22	0.87	0.005	0.004	−0.7	−0.15
	M	18	68	0.92	0.016	0.013	−0.4	0.3
NSC	F	31	23	0.9	0.005	0.003	−1.75	−1.48
	M	7	50	1.0	0.017	0.014	−0.86	−0.87
ATL	F	51	33	0.89	0.007	0.003	−1.7	−1.16
	M	25	94	0.96	0.02	0.014	−1.07	−0.78
ALE	F	32	47	0.95	0.01	0.007	−1.75	−0.61
	M	25	106	1.0	0.023	0.01	−1.9*	−1.9*
JSE	F	26	36	0.96	0.008	0.006	−1.89*	0.76
	M	15	70	1.0	0.017	0.01	−2.13**	−1.73
VAN	F	30	77	1.00	0.017	0.007	−2.13*	−1.85*
	M	18	105	1.0	0.025	0.015	−1.57	−1.52
PAC	F	88	121	0.99	0.021	0.008	−2.4**	−1.61
	M	58	212	1.0	0.039	0.013	−2.41**	−2.13*
Total	F	139	137	0.98	0.022	0.006	−2.46**	−1.7
	M	83	252	1.0	0.043	0.014	−2.32**	−2.06*

*N* number of individuals, *S* number of segregating sites, *HD* haplotype diversity, *θ* theta (per site), *π* nucleotide diversity per site with the Jukes and Cantor correction, *D*_0_, *D*_4_ Tajima’s *D* values for nondegenerate and fourfold degenerate sites, *nf* not found, *na* not available

* *P* < 0.05; ** *P* < 0.01; *** *P* < 0.001

**Fig. 2:**
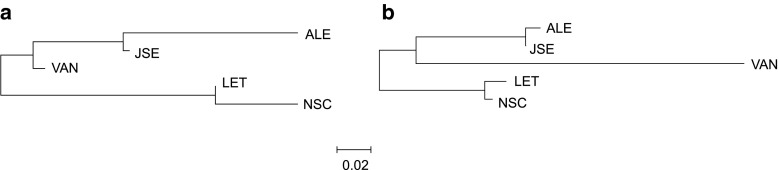
Neighbour-joining trees of sampled populations based on Φ_ST_ distances (Table 3) for F (**a**) and M (**b**) data

The AMOVA analysis of population structure pointed at the intrapopulation level of genetic differentiation as the main component of diversity for all studied samples (approximately 80 %). Various grouping of populations did not change this parameter substantially (Table 2). Despite the small share of interpopulation diversity, population pairwise comparisons (Table 3) showed significant differentiation. The only nondifferentiated pairs were the two Atlantic populations (NSC and LET) in both genomes, as well as the Pacific ALE and JSE populations (in M comparison only). The overall relationships among *M. trossulus* populations for the M and F lineages were reconstructed based on genetic distances between samples (Fig. 2). The topology of both trees was identical, and the overall length of the trees was similar, indicating the common history of both investigated markers. The only differences were in the metrics of the tree: some branches were disproportionate, the one leading to the VAN population was much longer in the M lineage than in the F lineage—a feature compatible with the pattern seen in Fig. 1, in particular with the distinct clade of M haplotypes unique to this sample. Not all branches within the M tree were longer than the corresponding F tree branches—a pattern somewhat surprising given the greater overall diversity within the M lineage.

**Table 2:** Hierarchical AMOVA in the M and F data sets

Genome	Sample grouping	*V*_a_ (%)	Φ_CT_	*V*_b_ (%)	Φ_SC_	*V*_c_ (%)	Φ_ST_
F	{VAN, JSE, ALE} {NSC, LET}	10.25	0.103	8.10	0.090*	81.65	0.184*
	{VAN} {JSE, ALE} {NSC, LET}	11.07	0.111	5.71	0.064*	83.22	0.168*
	{VAN} {JSE} {ALE} {NSC, LET}	16.77	0.168	−0.90	−0.011	84.13	0.159*
M	{VAN, JSE, ALE} {NSC, LET}	6.36	0.064	11.74	0.125*	81.90	0.181*
	{VAN} {JSE, ALE, NSC, LET}	15.66	0.157	7.41	0.088*	76.93	0.231*
	{VAN} {JSE, ALE} {NSC, LET}	17.69	0.177	0.75	0.009	81.56	0.184*

*V* variance components, *a* among groups, *b* among populations within groups, *c* within populations, Φ_CT_, Φ_SC_, Φ_ST_ fixation indices

* *P* < 0.01 following the Bonferroni correction for multiple tests

**Table 3:** Population pairwise Φ_ST_ values based on the *ND2*–*COIII* mtDNA region of the F (above diagonal) and the M (below diagonal) genomes for studied *Mytilus trossulus* samples

	LET	NSC	ALE	JSE	VAN
LET		0.009	0.209*	0.127*	0.111*
NSC	0.016		0.278*	0.183*	0.163*
ALE	0.126*	0.139*		0.094*	0.119*
JSE	0.142*	0.138*	0.008		0.079*
VAN	0.230*	0.208*	0.213*	0.196*	

* *P* < 0.05 following a Bonferroni correction for multiple tests

To investigate the influence of geographical distance on the observed genetic structure, the Mantel test was applied. The matrix of geographical distances, measured on the map as the shortest along-the-shore distances between samples, was correlated with the matrix of genetic distances in ARLEQUIN. No significant correlation was found in either the M (*P* = 0.30) or the F (*P* = 0.11) data set; the determination of genetic distance by geographical distance (*R* ^2^) was 21 % for the F data set and 22 % for the M data set.

### Demographic history

**Fig. 3:**
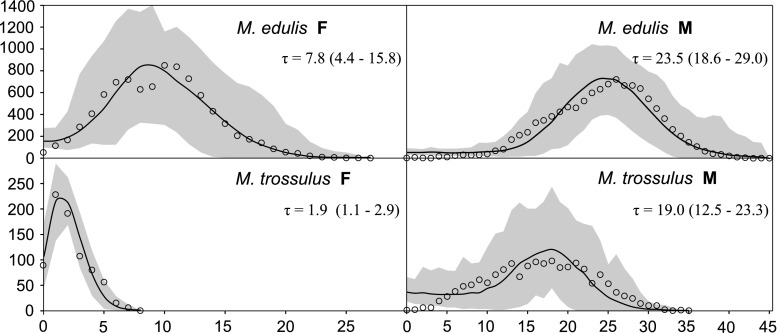
Mismatch analysis of selected groups of sequences. The data from Śmietanka et al. (2009) were analysed in the *upper panels*. Two well-defined Atlantic clades which experienced clear postglacial expansion were selected for this to serve as a reference point. For the *M. trossulus* data sets, two examples are presented in the *lower panels*. For the F genome, the small clade represented almost exclusively by Atlantic haplotypes with the characteristic *star*-like topology of several singleton haplotypes surrounding the central, more frequent (15 observations) haplotype (Fig. 1) was analysed. For the M genome, the whole Pacific data set was combined and analysed. In both cases, the inferred expansion is significantly more recent in the *M. trossulus* data set

The Tajima’s *D* statistics calculated separately for nondegenerate and fourfold degenerate sites (Table 1) indicated an excess of low-frequency polymorphisms producing the negative *D* values for both genomes, suggesting recent demographic expansion. To further investigate this possibility, the mismatch analysis was performed on selected groups of sequences. The sudden expansion model was fitted to the data sets in ARLEQUIN. In only a few cases, the fit was reasonably good. The overall M Pacific data set (all three Pacific samples combined) and the F Atlantic-specific clade produced the best fit to the model (Fig. 3, lower panels), confirming the suggestion that their negative *D* indices may be due to demographic reasons. The data from Śmietanka et al. (2009) were analysed in an analogous way to compare the timing of the events (Fig. 3, upper panel). In both cases, the timing of the expansions within the *M. trossulus* data sets was more recent than the one recorded in the Atlantic *M. edulis* data set. Moreover, within the *M. trossulus* data set, the expansion of the chosen F clade clearly post-dates the expansion of the M genome in the Pacific (two lower panels), when the differences in substitution rates between M and F genomes are taken into account.

**Fig. 4:**
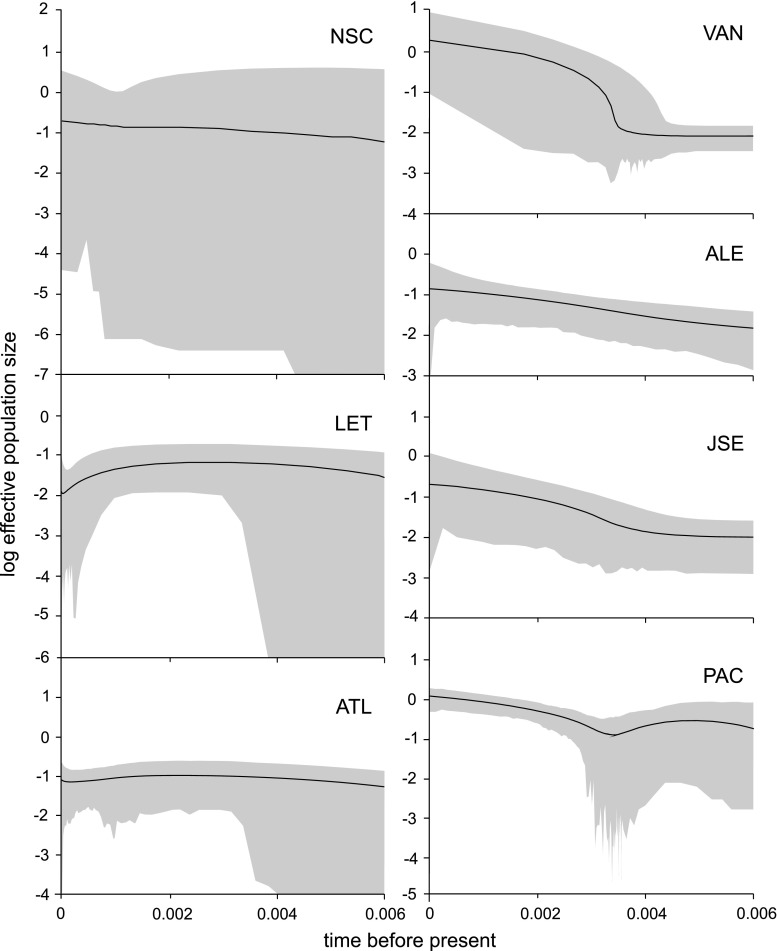
The examples of EBSP reconstructed for sampled populations of *M. trossulus.* All seven populations are shown. The three Pacific ones gave similar overall pattern of population growth. The combined ocean data sets were analysed separately, but, due to the evident population differentiation, the combined Pacific analysis may be biased and should be taken with caution. In the combined Atlantic data set, no clear population growth trend is visible

The unique presence of two independent mitochondrial markers with apparently similar genetic history permitted the application of a common demographic model while maintaining separate molecular evolution parameters for each marker. The reconstruction of past demography was attempted in BEAST, using the EBSP approach. The amount of diversity retained in population data sets was high enough to produce qualitative results only, with very broad confidence intervals, particularly for more distant events, consistent with a very shallow genealogy. This was the case also for the Atlantic data set (Fig. 4), even when both samples (NSC and LET) were combined, as seemed appropriate based on population pairwise comparisons (Table 3). In all the Pacific samples, continuous exponential growth throughout the depth of the genealogy was seen, whereas the pooled Atlantic group was rather stagnating within the same time frame, with possible, very recent fluctuations.

**Fig. 5:**
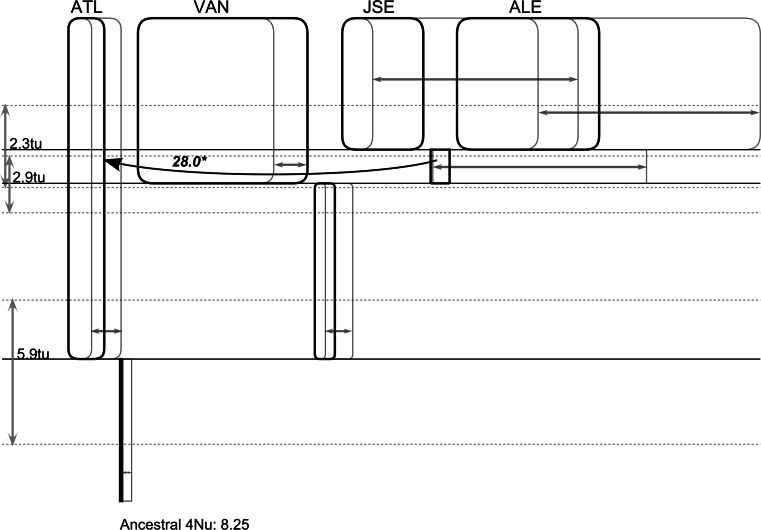
The model of population differentiation with potential migration was applied to the whole data set. To minimise the number of estimated indices, the two Atlantic samples were combined for this analysis. Despite that, not all parameters could be reliably estimated, in particular the posterior distribution of effective populations size measure (*q* = 4 Nu) for VAN was flat. However, the remaining indices can be trusted, since they apparently did not vary with widely changing priors on *q*. The presented *graph* was generated by IMfig (Hey 2010). The *vertical axis* represents time in mutational units; the unit represents the time in generations multiplied by the substitution rate (tu). The *horizontal axis* represents effective population size, again, in mutational units (*q*)—the unit represents four times the effective population size multiplied by the same substitution rate. The *bold*, *single*-*headed arrow* indicates an inferred significant migration event; the *number over the arrow* represents the number of migrants per generation (effective population size multiplied by the migration rate, Nm). The *two*-*headed arrows* indicate the confidence intervals around estimated effective population sizes (*q*) and divergence times (tu)

**Fig. 6:**
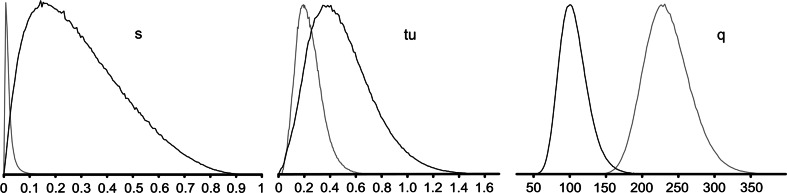
Comparison of two mussel invasions. The IM model was applied to two cases: *M. galloprovincialis* from Mediterranean Sea and Korea (*grey lines*), and *M. trossulus* from both sides of the Atlantic (NSC and LET samples—*black lines*). The restricted model with the single final effective population size parameter (*q* = 4 Nu) and no migration, but including the population split parameter (s) was run. The *plots* show posterior distributions of the three parameters, including the time of divergence in mutational units (tu). The *vertical axes* of all *plots* were normalised to the maximum value of each parameter; they measure the frequency with which particular values of the estimated parameters appear in the sampled set of genealogies

The EBSP approach typically assumes no genetic structure; therefore, the BSP obtained may be affected, if, in fact, such a structure exists, even in the Atlantic. The better model suitable for estimating the timing of differentiation and population size changes is implemented in IMa2. This software requires arbitrary decision on the population tree used. Several different trees were tried, but eventually the one suggested by ARLEQUIN was used (Fig. 2), as it was the most plausible one. The five population model could be implemented in IMa2, but with only two independent loci, it would be unlikely to produce meaningful estimates of all relevant indices. Therefore, we tried to implement the models restricted to as few parameters as possible. The pairwise comparisons of all samples with the full model indicated possible cases of nonzero migration parameters after the isolation. Based on these comparisons, the model with appropriately formulated priors and with the pooled Atlantic population produced the scenario with two well-separated isolation events, and a rather restricted case of potential transoceanic migration just after the second isolation event (Fig. 5). Notably, the ancestral population size inferred by IMa2 was inevitably very small. Even though the simple model implemented in ARLEQUIN indicated no genetic differentiation between Atlantic samples, there was a clear difference in genetic diversity (Table 1), suggesting certain asymmetry. Since the model implemented in IMa2 does not fit the scenario of asymmetric splits followed by potentially rapid population size changes, the older and slower IM had to be used, as it allows for the estimation of the population split parameter *s*. This parameter indicates how much the ancestral population contributed to each of the daughter populations. Several models were applied to the two Atlantic populations in IM, however, only the simplest one with no migration and a single population size parameter produced reliable results (Table 4). To compare these results with the well-known case of human mediated, very recent introduction of similar mussel species, the case of north-west Pacific *M. galloprovincialis* was used. The timing of this event is relatively well established at no more than 80 years before present (Wilkins et al. 1983). Other similar events are known, such as the introduction of *M. galloprovincialis* to South Africa or to the East Pacific coasts of North America. However, the South African invasion is considerably more recent (Grant and Cherry 1985) and less documented, whereas the North American case represents secondary invasion originating from Japan (Geller et al. 1994). Therefore, *M. galloprovincialis* mussels from NW Pacific represent one of the best examples of a human-mediated invasion of mussel and as such can be used as a reference case. The sequences of Korean mussels were compared with the sequences of Mediterranean mussels published by Śmietanka et al. (2009). The two closest samples were selected and combined as the sister population (samples ORI and GER from the original paper). This reference *M. galloprovincialis* data set was then analysed in IM using the same model as in the Atlantic *M. trossulus* case (Table 4). The obtained distribution of posterior probabilities (Fig. 6) indicates that the LET sample is derived from a rather larger fraction of the ancestral population than the BUS sample. Also, the time since isolation is most likely shorter in *M. galloprovincialis* case. Notably, the effective population size of *M. galloprovincialis* is definitely larger than that of *M. trossulus*.

**Table 4:** Comparison of IM runs for *M. trossulus* and *M. galloprovincialis*

Samples	*s*			tu			*q*		
	Est	LO	HI	Est	LO	HI	Est	LO	HI
BUS–MED	0.02	0.00	0.06	0.24	0.07	0.44	233	174	297
LET–NSC	0.23	0.02	0.63	0.43	0.08	0.98	105	70	145

95 % confidence intervals are shown along with the estimated value of three indices: population split parameter *s*, time since the divergence tu and the effective population size parameter *q*

### Tests of selection

Tests of selection were performed separately for M and F data sets. For verifying the relative pressure of purifying selection, coding parts of sequences were analysed. Diversity in synonymous and nonsynonymous substitutions was calculated (Table 5). The pattern of more relaxed selection within the M lineage evidenced by typically higher *π*_A_/*π*_S_ ratios was interrupted by apparently similarly lowered pressure in Atlantic F samples, with the F sample from LET having the *π*_A_/*π*_S_ ratio at the level of 0.236—the highest among all samples, both M and F. M–K tests were performed for several sequence groupings. With the very shallow genealogy and the lack of well-supported clades, there were no fixed differences at the intralineage level preventing application of the test. Analysis was possible for interlineage (M–F) comparison as well as for interspecies, intralineage comparisons, with the congeneric sequences of *M. edulis* and *M. galloprovincialis* taken from Śmietanka et al. (2009) (Table 6). For almost all comparisons, the P/F ratio in nonsynonymous substitutions was lower than in the synonymous ones, but statistical significance was observed only for interspecies M comparisons. In both cases, higher than expected fixation of replacement substitutions was observed.

**Table 5:** Jukes–Cantor corrected estimates of nonsynonymous (*π*_A_) and synonymous (*π*_S_) substitutions for intra-grouping comparisons in female (F) and male (M) genomes

Samples	*π*_A_ ± SE	*π*_S_ ± SE	*π*_A_/*π*_S_
F			
NSC	0.00081 ± 0.0003	0.00627 ± 0.0020	0.129
LET	0.00220 ± 0.0008	0.00934 ± 0.0028	0.236
VAN	0.00214 ± 0.0008	0.02152 ± 0.0034	0.099
JSE	0.00076 ± 0.0003	0.02159 ± 0.0046	0.035
ALE	0.00116 ± 0.0004	0.02402 ± 0.0052	0.048
ATL	0.00139 ± 0.0005	0.00746 ± 0.0022	0.186
PAC	0.00147 ± 0.0005	0.02436 ± 0.0040	0.060
Total	0.00148 ± 0.0004	0.01976 ± 0.0035	0.075
M			
NSC	0.00498 ± 0.0014	0.03712 ± 0.0064	0.134
LET	0.00579 ± 0.0015	0.03275 ± 0.0051	0.177
VAN	0.00511 ± 0.0013	0.03998 ± 0.0052	0.128
JSE	0.00465 ± 0.0011	0.02441 ± 0.0038	0.190
ALE	0.00353 ± 0.0009	0.02579 ± 0.0036	0.137
ATL	0.00577 ± 0.0014	0.03377 ± 0.0053	0.171
PAC	0.00498 ± 0.0009	0.03339 ± 0.0041	0.149
Total	0.00553 ± 0.0010	0.03559 ± 0.0036	0.155

*SE* standard error after computed by Bootstrap of 500 replicates

**Table 6:** Number of fixed (F) and polymorphic (P) synonymous and nonsynonymous substitutions for compared groups of *Mytilus* taxa

Compared taxa	Substitutions				
	F	P	P/F	*P*	*π*_S_ ± SE
TRO F–M					
Nonsynonymous	71	109	1.54	0.123	0.683 ± 0.073
Synonymous	77	163	2.12		
TRO F–EDU F					
Nonsynonymous	12	60	5	0.118	0.691 ± 0.078
Synonymous	67	189	2.82		
TRO M–EDU M					
Nonsynonymous	51	94	1.84	<0.0001	1.075 ± 0.137
Synonymous	66	304	4.61		
TRO M–GAL M					
Nonsynonymous	66	77	1.17	<0.001	1.168 ± 0.137
Synonymous	98	233	2.38		

*EDU F* CladeF1, *EDU M* CladeM1, *GAL M* CladeM2 from Śmietanka et al. (2009)

*P* probabilities from two-tailed Fisher’s exact test

## Discussion

The observed pattern of polymorphism suggests that the world population of *M. trossulus* is very young. This species is regarded as the oldest one of the *M. edulis* species complex, consistent also with the mitochondrial genealogy (Riginos and Cunningham 2005; Śmietanka et al. 2010; Zbawicka et al. 2010). Consequently, it should have larger intraspecies genetic diversity than the younger congeners. Contrary to that expectation, the overall level of nucleotide diversity (Table 1) within the whole data set covering the widest geographical area possible for *M. trossulus* is comparable with the diversity seen in single *M. edulis* clades (Śmietanka et al. 2009).

In *M. edulis* reciprocal, monophylly of two very divergent M genomes is observed in transatlantic comparisons (Riginos et al. 2004). According to Śmietanka et al. (2010), the two *M. edulis* M haplotypes diverged a few 100,000 years ago, clearly well before the last glacial maximum (LGM). Contrary to that, in *M. trossulus,* no such phenomenon is seen. The ancestral position of *M. trossulus* in respect to *M. edulis* and *M. galloprovincialis* is unquestionable, so the low observed diversity must have resulted from bottleneck events at LGM. Comparison of mismatch analyses suggests that the expansion events within *M. trossulus* populations were much more recent than the postglacial expansion of *M. edulis* in the Atlantic (Fig. 3). The *τ* values estimated from this mismatch analysis can be used to date the demographic events inferred by IM, IMa2 and BEAST (Figs. 4, 5 and 6). Both coalescence-based approaches (IMa2 and BEAST) estimated the relative substitution rate in M and F genomes at approximately 2.5, the relative *τ* values derived from the mismatch analyses presented in Fig. 3 are also comparable. If we assume that the *τ* of approximately 8 obtained for the Atlantic *M. edulis* F clade (Fig. 3, upper left) corresponds with the onset of expansion at the end of LGM (Clark et al. 2009), no earlier than 18,000 years ago (kya) then the F substitution rate would have to be no less than 2.2 × 10^−4^. In IMa2, the geometric mean of substitution rates is used; therefore, with the M substitution rate at 2.5 times the F substitution rate, the mean substitution would then be approximately 3.5 × 10^−4^. This value could be used to convert indices obtained in IMa2 simulations (Fig. 5) to demographic and absolute time units: the trans-oceanic expansion would have to occur no earlier than 16.8 kya, and the split of Pacific populations accompanied by the second transoceanic migration at approximately 8 kya. This is consistent with the Bering Strait opening at 10 kya and subsequent persistence of mussels in the Arctic, in agreement with the paleontological evidence (Feder et al. 2003). The effective population size of *M. trossulus* at LGM would have to be a few thousand individuals only (approximately 6,000). In an analogous way, the scale in Fig. 4 could be converted to the same absolute time units. In this case, the M lineage substitution was used as the reference and per site (as opposed to per-locus) substitution was applied, therefore, the value of 0.006 in mutational units corresponds to the time of approximately 13 kya. This indicates that the extant populations of *M. trossulus* retained the information on the postglacial events only, and that probably only a single and very small refugium of these mussels survived LGM. Consequently, they have lost almost all the diversity pre-dating the bottleneck. This is consistent with the notion of postglacial Atlantic colonisation postulated by several authors (Rawson and Harper 2009; Väinölä and Strelkov 2011), but extends the idea to the whole species’ range. The difference between the EBSP (Fig. 4) results for mussels from the two oceans—exponentially growing population in the Pacific but somewhat restricted and stagnating populations in the Atlantic—would have resulted if the *M. trossulus* mussels in the Atlantic met the native *M. edulis* which survived the LGM in the Atlantic. The resulting competition may have restricted the expansion. The inevitable hybridisation could have led to the observed disruption of DUI—specific *π*_A_/*π*_S_ patterns (Table 5). This scenario may seem unreasonable, since the observed genetic diversity (particularly HD—Table 1) is very high, which is unexpected so shortly after the extreme bottleneck. But, given a high enough substitution rate, it is entirely possible to recreate this level of polymorphism. In fact, the extreme bottleneck coupled with rapid expansion may have facilitated the onset of the compensation draft feedback (CDF) process, as postulated by Śmietanka et al. (2010), resulting in rapid recreation of the lost diversity, and the evident signature of positive selection within one of the lineages (Table 6). The observed signature of positive selection could not possibly be the artefact resulting from a saturation effect. The saturation may have affected the results—the nucleotide diversity at synonymous sites for the most divergent groups of sequences is above 1 (Table 6, last column). However, the direction of this effect would be opposite the observed one. It would have caused underestimation of the number of silent substitutions, possibly increasing the number of polymorphic ones and decreasing the number of fixed ones. It is unlikely that the number of nonsynonymous substitutions would be equally affected; hence, the imbalance between the ratios observed in Table 6 (P/F column) would be even greater.

The origin of *M. trossulus* at Loch Etive is most likely transatlantic but it is unlikely that their invasion was human mediated. The comparison with the well-documented human-mediated introduction of *M. galloprovincialis* to Asia, which occurred no later than 100 years ago, suggests that the transatlantic invasion of *M. trossulus* must have occurred earlier. If the same substitution rate was to be used for dating, the value of 0.4 tu (Fig. 6) would correspond to more than 1,000 years. This estimate must be taken with caution as time-dependent substitution rate variation (Ho et al. 2005) will most likely lead to problems when the dated events are so recent. There is another, more striking difference between the two mussel invasions. The share of the ancestral population in the invasive one was estimated at a very small value (approximately 2 %) for *M. galloprovincialis* case, whereas the marginal density of the *s* parameter for *M. trossulus* case, although much wider, had the maximum at appropriately 20 %. Such a large value is inconsistent with human-mediated transport of a small number of individuals. Therefore, it can be concluded that the most likely source of European *M. trossulus* is the North American Atlantic population, and that this invasion occurred relatively recently, within the time frame short enough for both mitochondrial genomes to retain the history of this event. Our estimates on *s* and tu may not be precise enough to state when exactly the split between Canadian and Scottish *M. trossulus* occurred, or to conclude with absolute certainty that the shares of the ancestral population are different in the compared cases, but, due to the overall high observed diversity (the LET sample has higher diversity even than the BUS sample, despite obviously lower effective population size—Fig. 6), it is unlikely that this invasion is an ongoing or human-mediated process. We have shown the fine phylogeographical events in *M. trossulus* to the extent possible with just the two independent mitochondrial markers. Some conclusions are very firm. There is no doubt that the source of the recent expansion of the species in Europe is Atlantic and not Pacific. There is also no doubt that the overall level of diversity is shaped almost exclusively by the extreme bottleneck at LGM, and this effect is much stronger in *M. trossulus* than in its congeners (Śmietanka et al. 2009). Some parameters, in particular the exact timing of the events, are, however, estimated very roughly, with broad confidence intervals, precluding very definite statements regarding the involvement of human-mediated transport in the very latest developments.

More markers (nuclear microsatellites or SNPs) are needed to resolve the remaining uncertainties, but the proposed scenario (Fig. 5) is nevertheless the most probable. The expansion from a single Pacific refugium started immediately after the LGM, and as soon as the sea level raised to the point of opening the Bering Strait, *M. trossulus* migrated to the Arctic and further to the Atlantic. These events were contemporary, with further spatial and demographic expansion in the Pacific during the climatic optimum at around 10 kya. Apparently, the genetic continuity within Atlantic lasted much longer than in the Pacific, leading to the lack of differentiation between *M. trossulus* mussels inhabiting west and east coasts of this ocean. This may have been caused by smaller physical distances and more favourable oceanic currents, apparently allowing long distance colonisation. The involvement of human-mediated transport is unlikely but requires further investigation.
